# Determine the Potential Epitope Based Peptide Vaccine Against Novel SARS-CoV-2 Targeting Structural Proteins Using Immunoinformatics Approaches

**DOI:** 10.3389/fmolb.2020.00227

**Published:** 2020-10-15

**Authors:** Muhammad Waqas, Ali Haider, Muhammad Sufyan, Sami Siraj, Sheikh Arslan Sehgal

**Affiliations:** ^1^Department of Bioinformatics and Biotechnology, Government College University Faisalabad, Faisalabad, Pakistan; ^2^Institute of Basic Medical Sciences, Khyber Medical University, Peshawar, Pakistan; ^3^Department of Bioinformatics, University of Okara, Okara, Pakistan

**Keywords:** immunoinformatics, SARS-CoV-2, SARS-CoV, peptide vaccines, corona virus disease 2019

## Abstract

Coronaviruses (CoVs) belong to the Coronaviridae-family. The genus Beta-coronaviruses, are enveloped positive strand RNA viruses with club-like spikes at the surface with a unique replication process and a large RNA genome (∼25 kb). CoVs are known as one of the major pathogenic viruses causing a variety of diseases in birds and mammals including humans (lethal respiratory dysfunctions). Recently, a new strain of coronavirus has been identified and named as SARS-CoV-2. A large number of COVID-19 (disease caused by SARS-CoV-2) cases are being diagnosed all over the World especially in China (Wuhan). COVID-19 showed high mortality rate exponentially, however, not even a single effective cure is being introduced yet against COVID-19. In the current study, immunoinformatics approaches were employed to predict the antigenic epitopes against COVID-19 for the development of a coronavirus peptide vaccine. Cytotoxic T-lymphocyte (CTL) and B-cell epitopes were predicted for SARS-CoV-2 coronavirus structural proteins (Spikes, Membrane, Envelope, and Nucleocapsid). The docking complexes of the top 10 epitopes having antigenic sites were analyzed led by binding affinity and binding interactional analyses of top ranked predicted peptides with the MHC-I HLA molecule. The predicted peptides may have potential to be used as peptide vaccine against COVID-19.

## Background

There are still a variety of human diseases with unknown etiology. A viral parentage has been purposed for numerous diseases which also has significance to search for new viruses (Cascella et al., 2020). However, there are various difficulties involved in scrutinizing new viruses, as some viruses do not replicate *in vitro* and have cytopathic effects (CPE). The viruses that are unable to replicate *in vitro* lead to the failure of virus discovery. The DNA Amplified Restriction Fragment Length Polymorphism (cDNA-AFLP 4) technique helps to identify new viruses, including the discovery of the new coronavirus (CoV) (Cascella et al., 2020). The SARS-CoV-2 strain from the genus Beta-coronavirus of the Coronaviridae family, are enveloped viruses with a large plus strand RNA genome, complete classification is provided in Supplementary Material. The size of the genomic RNA is 27–32 kb and poly-adenylated. There are three serologically distinct groups of CoVs. Viruses are characterized by their genomic sequence and host range (Guy et al., 2000). CoVs have been discovered in mice, turkeys, cats, horses, and humans, leading to many diseases including respiratory tract issues and gastroenteritis (International Committee on Taxonomy of Viruses, 2020). Two human viruses (HCoV-229E and HCoV-OC43) were identified in the mid-1960s and are known to cause the common cold. The recently identified SARS-CoV-2 causes a life-threatening pneumonia and is the most pathogenic human CoV identified thus far (Peiris et al., 2003). SARS-CoV-2 is likely to have been occupied in an animal source and recently initiated the pandemic in humans through zoonotic transmission (Martina et al., 2003). SARS-CoV-2 is the first member of a fourth group of CoVs (Snijder et al., 2003).

In Wuhan (Hubei Province, China), a number of patients linked with Hunan South China seafood market have the third zoonotic human CoV of the century which emerged on the 31st of December, 2019. CoV is similar to Severe Acute Respiratory Syndrome coronavirus (SARS-CoV) and Middle East Respiratory Syndrome Coronavirus (MERS-CoV) infections including fever, lung infiltration and difficulty in breathing (de Wilde et al., 2018; Wuhan Municipal Health Commission, 2020). After an extensive speculation about the causative agent of CoV, the identification of the novel CoV was announced by the Chinese Center for Disease Control (CDS) on the 19th of January, 2020 (Kahn, 2020). The novel CoV SARS-CoV-2 was insulate from a single patient and later corroborated by 16 more patients (World Health Organization [WHO]., 2020). The viral pneumonia of COVID-19 was quickly predicted as a likely causative agent and the sequence of SARS-CoV-2 was submitted (VoNCGAohvotn-c-gaoJ, 2020). Later, five more sequences of SARS-CoV-2 were submitted on the GSAID database on 11th of January, 2020 from the Chinese institutes (GDCAohwgoCaoJ, 2020). Multiple sequence alignment of SARS-CoV, MERS-CoV, and SARS-CoV-2 was carried out and the conserved part of DNA and protein sequences was observed to be similar. Hundreds of deaths linked with this deadliest infection increase the morbidities in the age of 50 years and above. Various diseases have been discovered and associated including dry-cough, leukopenia, fever, and shortness of breath. The extracorporeal membrane oxygenation of the patients considered as severe cases need supportive care. The infection of SARS-CoV-2 in elderly patients is known to be less virulent as compared to SARS-CoV (10% mortality) and MERS-CoV (35% mortality) in the initial stage, later on SARS-CoV-2 caused a huge mortality rate in all over the world (Imai et al., 2020). For this infection, no reliable mediation is currently available. Preventative measures are urgently needed due to the significant global disease burden resultant of SARS-CoV-2 (Douglas et al., 2018). SARS-CoV-2 has a far higher mortality rate as compared to the other known members of corona virus family and researchers are trying their best to develop a successful vaccine against COVID-19. Peptide-based vaccines and multi-epitope adjuvant based vaccines approaches (Tahir ul Qamar et al., 2020) are used widely for the development of successful vaccine. Moreover, naturally occurring compounds are also employed to inhibit SARS-CoV-2 efficiently by using virtual screening approaches (Xiao et al., 2020).

The vaccine development process essentially involves the determination of effective B-cell epitopes and Cytotoxic T lymphocytes (CTL). The advanced methodology has emerged to determine the response of T-cells against numerous vaccine candidates for the process of vaccine development (Ip et al., 2015). The present effort struggles to elucidate and scrutinize the effective T-cells and B-cell (conformational and linear) epitopes act as potential candidates for vaccine by utilizing the immunoinformatics approaches. Furthermore, the crucial step for the development of a vaccine is the identification of potential peptides from the virulent pathogen proteome having interactions with the major histocompatibility complex (MHC). The efficiency of the epitopes binding to MHC molecules is linked with the T-cell immunogenicity (Lazarski et al., 2005). An immunoinformatics approach was utilized to predict the peptide-MHC complexes and comparative molecular docking analyses leads to scrutiny of the potential peptides for peptide vaccine development. Recently, similar approaches and methodology were used against Zika virus, MERS-CoV virus, and Ebola virus for peptide-based vaccine prediction (Ashfaq and Ahamed, 2016; Ahmad et al., 2019; Tahir ul Qamar et al., 2019a).

## Materials and Methods

### Sequence Retrieval

The primary amino acid sequences of the structural proteins of CoV were extracted from NCBI (Geer et al., 2010). The amino acid sequences of the selected structural protein of CoV have 222 residues for membrane protein (NCBI_Protein = QHQ82467.1), 75 residues for envelope protein (NCBI_Protein = QHW06051.1), 419 residues for nucleocapsid protein (NCBI_Protein = QHZ00386.1) and 1273 amino acids for spikes protein (NCBI_Protein = QHR63260.2). The physiochemical properties of the selected protein were evaluated by using Protparam and VOLPES (Wilkins et al., 1999).

### Multiple Sequence Alignment (MSA)

Multiple Sequence Alignment was performed on all the three full length genomes (SARS-CoV = NC_004718, MERS-CoV = NC_019843.3 and SARS-CoV-2 = NC_045512.2) and the genomic sequences were retrieved through GenBank (Sayers et al., 2019, 2020). The genomic sequences of the selected genomes were utilized and a hierarchical approach along with a series of different pair-score matrices including sum-of-pairs and Hidden Markov Model (HMM) was employed for MSA. Clustal Omega (Sievers and Higgins, 2014, 2018) was utilized to analyze the MSA of the selected genomic sequences and the conserved domains were observed by using WebLogo3 (Crooks et al., 2004).

### Conformational and Linear B-Cell Epitopes Prediction

The antigen B-cell epitope interactions against B-lymphocyte leads to the differentiation of B-lymphocytes into two different types of cells as antibody-secreting plasma and memory cells (Nair et al., 2002). The hydrophilic nature and surface accessibility of B-cell epitopes were assumed as the key characteristics of predicted B-cell epitopes as predicted B-cells epitopes should be water loving in nature for better solubility (Parker et al., 1986) by accessing the immune epitope database and analysis resource (IEDB)^1^ as stated by hydrophilicity prediction of Parker (Parker et al., 1986), flexibility prediction of Karplus and Schulz (1985), Emini surface accessibility prediction (Pettersen et al., 2004) and antigenicity scale of Kolaskar and Tongaonkar (Alexander et al., 2011). The conformational B-cell epitopes were predicted by employing ElliPro^2^ (Pettersen et al., 2004) from IEDB analysis resource having three diverse algorithms comprising protein shape approximation (Emini et al., 1985), residues Protrusion Index (PI) (Nain et al., 2019) and the adjacent residues clustering based on PI.

### Potential Epitope Prediction of Cytotoxic T-Lymphocyte (CTL)

The CTL epitopes predictions were analyzed through utilizing NetCTL.1.2 server (Beijing News, 2020). The molecules of MHC behave as antigens and utilize their surface for the activation of CTLs. The NetCTL.1.2 server was utilized to integrate the MHC class I binding prediction, proteasomal C-terminal cleavage and transporter associated with antigen processing (TAP) transport efficiency. The FASTA format sequences of the organism were subjected to the server and Human leukocyte antigen (HLA) alleles and peptide lengths were observed and analyzed. Additionally, the prediction of T-cell epitopes and weight matrix algorithm was employed for the prediction of TAP transport efficiency and artificial neural network was implemented to predict the MHC class-I binding and proteasomal C-terminal cleavage.

### World Population Coverage Analyses

The World population coverage analyses were performed by utilizing the IEDB server. The selected CTL epitopes were used and analyzed against the respective allele sets and major world populations were covered. The key purpose of the coverage analyses was to analyze whether the selected candidates were suitable for major populations or not. The analyses were performed against China, Iran, Japan, Korea, Pakistan, Italy, France, and other countries which are being affected by SARS-CoV-2 in the 2020 viral outbreak (Vita et al., 2019).

### Molecular Docking Analyses and Peptide-MHC Protein Complex

The predicted epitopes of SARS-CoV-2 structural proteins with antigenic residues were selected for molecular docking analyses. The PEP-FOLD3 server (Lamiable et al., 2016) was utilized to predict the 3D structures of the selected peptides with 200 simulation runs to sample the conformations. The conformational models clustered by the PEP-FOLD3 server were evaluated on the basis of sOPEP energy scores (Maupetit et al., 2007). The analyzed peptides which had higher scores were selected for molecular docking experiments with MHC class I binding molecule comprising HLA-B (PDB ID: 3VCL) through PatchDock docking server (Huang et al., 2010). All the docked complexes having undesirable penetrations of the receptor’s atoms into the ligand were rejected and geometric shape complementarity score was applied to classify the other complexes. Subsequently, the FireDock server (Andrusier et al., 2007; Mashiach et al., 2008) was utilized to refine the docked complexes and also predict the score of the docking outputs.

**FIGURE 1:**
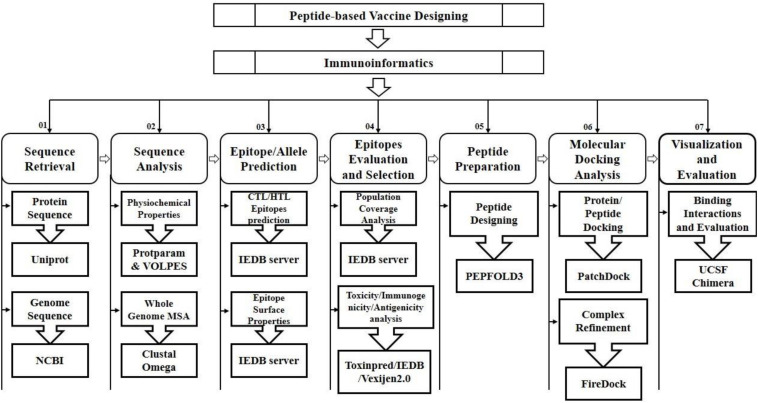
Schematic workflow for the prediction of peptide based vaccine against SARS-CoV-2.

The FireDock server was utilized to improve the flexibility and scoring errors observed during the molecular docking calculations through fast rigid-body docking tools (Kingsford et al., 2005). The molecular visualization programs PyMOL (Alexander et al., 2011), Ligplot and UCSF Chimera 1.11 (Pettersen et al., 2004) were utilized to visualize, analyze and identify the hydrogen bonding interactions of the docked complexes (Nair et al., 2002; Palatnik-de-Sousa et al., 2018; Tahir ul Qamar et al., 2019b). The schematic diagram illustrating the applied approaches and strategies along with tools and software are mentioned in Figure 1.

## Results

A variety of tools and servers have resulted through recent advancements in immunological bioinformatics, which lessen the time and cost of traditional vaccine advancement. The development of an effective multiple epitope vaccine remains difficult due to problems in selection of suitable antigen candidates and immune-dominant epitopes. Thus, it is important to predict the appropriate antigen epitopes of the targeted protein by immune-informatics approaches to design a multiple epitope vaccine (Nain et al., 2019). The main target was to use the immune-informatics approaches and the prediction of peptide vaccine through recognizing MHC binding, B-cells and CTL epitopes. The discovery of effective vaccines is possible through pathogenomics analyses on a genome wide scale, though these conventional experimental methods have multiple limitations (Rodrigues et al., 2019). Immune-informatics approaches help to analyze the complete spectrum of the potential antigen, and furthermore complications regarding *in vitro* expression of antigen and pathogen culturing can also be evaded. By means of computational methods, the immune research groups have reported various vaccine candidates as having promising preclinical outputs (Davies and Flower, 2007). In current efforts, epitopes have been identified to design the peptide vaccine against HLA-B protein (Tahir et al., 2018). The development of epitopes based vaccines targeting the structural proteins of SARS-CoV-2 and epitopes of the target proteins were predicted to support the host’s immune response. The antigenicity and allergenicity of the predicted epitopes were observed through VaxiJen and Allergen F.P 1.0 (Dimitrov et al., 2014). The estimation of population coverage of predicted epitopes was calculated and it was observed that the coverage in China was 0.5639 with average hits of 4.0 for MHC class I, and with average 0.2462 and hits of 0.91 for MHC class II (Supplementary Table 1). The peptides were designed against ten epitopes by utilizing Pepfold-3.0. The molecular docking analyses of the selected ten peptides were performed through PatchDock and further refined through Fire Dock (Andrusier et al., 2007; Mashiach et al., 2008; Huang et al., 2010) to identify the effective binding sites (Table 1).

**TABLE 1:** Predicted CTL epitopes from the SARS-CoV-2 structural proteins having antigenic sites.

Residue number	Peptide sequence	Predicted MHC binding affinity	Rescale binding affinity	C-terminal cleavage affinity	TAP transport efficiency	Prediction score
**Spikes**						
865	LTDEMIAQY	0.7953	3.3768	0.9723	2.779	3.6616
258	WTAGAAAYY	0.6735	2.8596	0.7339	2.863	3.1128
604	TSNQVAVLY	0.6559	2.7847	0.944	2.991	3.0758
361	CVADYSVLY	0.5348	2.2705	0.9764	3.18	2.5759
733	KTSVDCTMY	0.4908	2.084	0.9649	3.016	2.3795
746	STECSNLLL	0.5136	2.1808	0.8879	0.703	2.3492
**Membrane**						
213	SSDNIALLV	0.6531	2.7729	0.9682	0.286	2.9325
171	ATSRTLSYY	0.5463	2.3195	0.9375	3.09	2.6146
**Envelope**						
34	LTALRLCAY	0.5594	2.3	0.672	2.933	2.6158
**Nucleocapsid**						
104	LSPRWYFYY	0.4837	2.0538	0.9746	2.815	2.3408

### Analysis for SARS-CoV-2 Structural Proteins Surface Properties

A peptide with surface-accessibility probability of >1.0 reflects more probable chances for a peptide to be found on the surface (Parker et al., 1986). Numerous peptides were predicted and the top ranked predicted peptides of SARS-CoV-2 structural proteins on the basis of surface probability (*Y*-axis) and sequence position (*X*-axis) were selected for further analyses (Supplementary File 1–4). The maximum surface probability scores for the membrane protein, envelope protein, nucleocapsid protein and spikes protein were analyzed as “YANRNR” 5.199, “YSRVKN: 4.136, “KKDKKK” 6.966, and “QDKNTQ” 6.051, respectively. Similarly, minimum surface probability scores for the membrane protein, envelope protein, nucleocapsid protein and spikes protein were observed as “LACFVL” 0.078, “LCAYCC” 0.088, “LALLLL” 0.05, and “VFLVLL” 0.07, respectively (Table 2).

**TABLE 2:** The maximum and minimum values of the predicted peptides.

Protein	Length	Molecular Weight (g/mol)	Hydropathicity	Theoretical PI	Max. surface accessibility (%)	Min. surface accessibility (%)	Max. Flexibility (%)	Min. Flexibility (%)	Max. Antigenicity (%)	Min. Antigenicity (%)
Membrane	222	25146.6	0.446	9.51	5.199	0.078	8.7	0.061	7.6	0.01
Envelope	75	83650.4	1.128	8.57	4.136	0.088	6.53	0.005	7.5	0.002
Nucleocapsid	419	45625.7	−0.971	10.07	6.966	0.05	8.8	0.052	6.925	0.05
Spikes	1273	141178	−0.079	6.24	6.051	0.07	5.7	0.3	6.121	0.0751

The Karplus and Schulz (1985) flexibility method was utilized to calculate and analyze the atomic vibrational motions in the protein structure designated through B-factor and temperature. The stability and organization of the structure depends upon the B-factor values. The quality of the predicted models depends upon the B-factor values as a lower B-factor value is considered as an effective model while higher B-factor values lead to the less organized and poorly ordered structures (Karplus and Schulz, 1985; Table 2).

The hydrophilicity scale process of Parker was carried out to observe the peptides hydrophilicity based on the peptide retention times through HPLC on reversed phase column. Immunological analyses have revealed the association of antigenic sites with the hydrophilic regions (Parker et al., 1986). The antigenicity of SARS-CoV-2 was calculated through the Kolaskar & Tongaonkar method (Table 2). The predicted facts and data for all selected four protein properties are mentioned in the Supplementary Material (Supplementary File 1–4).

### Structure-Based Epitope Prediction for SARS-CoV-2 Structural Proteins

The correlation among the protein structure antigenicity, epitope prediction, accessibility and flexibility within 3D structures were determined through ElliPro (Ponomarenko et al., 2008). The significant properties including protein-antibody interactions were analyzed to differentiate the predicted epitopes. The top-ranked five conformational epitopes for SARS-CoV-2 which had a score of ≥0.6 were observed and selected for further analyses. The PI (Isoelectric Point value) (Ponomarenko et al., 2008) score was observed to analyze the percentage of the atoms which extend over the molecular bulk and are also liable for the antibody binding. The top ranked 2 conformational predicted epitopes along with the residues name, length and locations were critically analyzed (Table 3) and the score was observed 0.703 and 0.706.

**TABLE 3:** Top ranked selected discontinues epitopes, interacting residues and scores.

Sr. No.	Residues	Number of residues	Score
**Predicted Discontinuous Epitopes**			
1	A:G11, A:K12, A:G15, A:C16, A:C22, A:G23, A:T24, A:W31, A:D33, A:D34, A:R40, A:C44, A:T45, A:S46, A:E47, A:D48, A:M49, A:L50, A:N51, A:P52, A:N53, A:Y54, A:E55, A:D56, A:L57, A:L58, A:I59, A:R60, A:K61, A:S62, A:N63, A:H64, A:N65, A:L67, A:Q69, A:A70, A:G71, A:N72, A:V73, A:Q74, A:L75, A:R76, A:V77, A:I78, A:G79, A:H80, A:S81, A:M82, A:K90, A:V91, A:D92, A:T93, A:A94, A:N95, A:P96, A:K97, A:T98, A:P99, A:K100, A:N133, A:D155, A:C156, A:G183, A:P184, A:F185, A:V186, A:D187, A:R188, A:Q189, A:T190, A:A191, A:Q192, A:A193, A:A194, A:G195, A:T196, A:D197	77	0.706
2	A:S1, A:G2, A:F3, A:T198, A:T199, A:V212, A:I213, A:N214, A:G215, A:D216, A:R217, A:W218, A:F219, A:L220, A:N221, A:R222, A:F223, A:T224, A:T225, A:T226, A:L227, A:N228, A:D229, A:F230, A:N231, A:L232, A:V233, A:A234, A:M235, A:K236, A:Y237, A:N238, A:Y239, A:E240, A:P241, A:L242, A:T243, A:Q244, A:D245, A:V247, A:D248, A:G251, A:P252, A:S254, A:A255, A:Q256, A:T257, A:G258, A:I259, A:A260, A:V261, A:L262, A:D263, A:A266, A:S267, A:K269, A:E270, A:L271, A:L272, A:Q273, A:N274, A:G275, A:M276, A:N277, A:G278, A:R279, A:T280, A:I281, A:L282, A:G283, A:S284, A:A285, A:L286, A:S301, A:G302, A:V303, A:T304, A:F305, A:Q306	79	0.703

### Molecular Docking Analyses of SARS-CoV-2 Structural Proteins With HLA-B

The comparative molecular docking analyses were executed for the top ranked 10 selected epitopes of SARS-CoV-2 out of 87 designed peptides with MHC class I HLA-B. The effective binding affinities have been observed for all the selected CTL epitopes having van der Waals (VdW) energy values ranges from −21.80 to −27.52 kcal/mol and the observed global energy was −25.01 to −53.65 kcal/mol (Table 4). The molecular docking analyses of the selected 10 CTL predicted epitopes were carried out and effective binding affinities with HLA-B were observed (Supplementary File 5).

**TABLE 4:** The designed peptides against SARS-CoV-2 peptides-MHC class I HLA-B interactions.

Peptide	Global energy (kcal/mol)	Attractive VdW energy (kcal/mol)	H bond Energy (kcal/mol)	Peptide-MHC pair	Bond distance (Å)	Conserved residues
LTDEMIAQY	−34.23	−26.46	−1.11	ILE 6 CD ALA 69.A CB	2.400	TYR9
				TYR 9 CB GLN 70.A OE1	2.306	ARG62
				ILE 6 CD ALA 69.A HB3	1.855	ILE66
				TYR 9 O1 GLN 70.A HE22	1.458	THR73
				MET 5 CE ARG 62.A NH1.B	2.738	TYR99
				TYR 9 O1 GLN 70.A NE2	2.350	GLU152
				GLN 8 NE2 GLN 155.A C	2.604	
WTAGAAAYY	−45.23	−26.05	−0.50	TYR 9 CB ALA 158.A CB	2.123	TYR9
				TRP 1 CZ3 ARG 62.A NH2.B	2.064	ARG62
				GLY 4 CA ILE 66.A CD1	3.964	ILE66
				TRP 1 CD1 TYR 7.A CD2	3.846	THR73
				TRP 1 CE2 ASN 63.A CB	3.797	TYR99
				ALA 5 CB TYR 159.A HD2	3.278	GLU152
TSNQVAVLY	−49.99	−26.52	−0.67	LEU 8 CD2 TYR 99.A CZ	2.524	TYR9
				GLN 4 NE2 ARG 62.A HE.B	1.596	ARG62
				GLN 4 NE2 ARG 62.A NH1.B	2.223	ILE66
				LEU 8 CD2 TYR 99.A OH	2.339	THR73
				LEU 8 CD2 TYR 99.A CE2	2.564	TYR99
						GLU152
SSDNIALLV	−40.24	−22.14	−1.32	LEU 8 CA ILE 66.A CG2	3.963	TYR9
				VAL 9 CG2 ARG 156.A NE	3.890	ARG62
				LEU 7 CB ILE 66.A HG13	3.269	ILE66
				LEU 8 CD2 ILE 66.A C	3.971	THR73
				ALA 6 CA TYR 159.A CB	3.973	TYR99
				ASP 3 C GLU 163.A CD	3.799	GLU152
				ASP 3 C ARG 62.A CZ.B	3.800	
LTALRLCAY	−53.65	−26.02	−1.97	THR 2 CB GLN 70.A NE2	3.881	TYR9
				THR 2 CG2 ALA 69.A CB	3.958	ARG62
				CYS 7 SG ARG 62.A NH2.B	3.773	ILE66
				LEU 1 CG TYR 116.A OH	3.763	THR73
				TYR 9 CE2 GLN 155.A OE1	3.625	TYR99
				LEU 4 CD1 ASP 114.A CG	3.969	GLU152
				ALA 3 CB GLN 70.A OE1	3.749	
ATSRTLSYY	−48.57	−27.52	−1.18	SER 6 CA ILE 66.A HG22	3.258	TYR9
				TYR 9 O1 GLN 70.A HE22	2.865	ARG62
				TYR 9 CB TYR 99.A CG	3.970	ILE66
				TYR 9 CD1 ARG 156.A HH12	3.150	THR73
				VAL 2 CA ARG 62.A HH22.B	3.271	TYR99
				TYR 9 CZ ARG 156.A CA	3.796	GLU152
				TYR 5 OH TYR 159.A CA	3.556	
CVADYSVLY	−38.79	−25.19	−2.05	THR 5 CG2 ARG 156.A CZ	3.943	TYR9
				ALA 1 N ALA 158.A HB3	3.004	ARG62
				TYR 9 CE2 TRP 147.A CH2	3.829	ILE66
				TYR 9 O1 TYR 9.A CE2	3.551	THR73
				ALA 1 CB GLN 155.A NE2	3.876	TYR99
				TYR 9 CE1 ARG 156.A HH21	3.136	GLU152
				ALA 1 CA GLN 155.A CG	3.956	
KTSVDCTMY	−27.73	−21.80	−2.62	LYS 1 CE GLN 70.A OE1	3.721	TYR9
				TYR 9 O2 THR 73.A CG2	3.542	ARG62
				MET 8 CE ARG 156.A NE	3.876	ILE66
				VAL 4 CG1 ILE 66.A H	3.254	THR73
				THR 2 CG2 TYR 159.A CG	3.968	TYR99
				VAL 4 CG2 ILE 66.A HG23	3.273	GLU152
				VAL 4 CA ILE 66.A HG12	3.274	
STECSNLLL	−45.10	−24.24	−2.18	SER 5 CA ARG 156.A CD	3.956	TYR9
				LEU 7 CD1 ALA 69.A CB	3.961	ARG62
				CYS 4 CB GLU 152.A OE2	3.745	ILE66
				SER 1 OG ALA 150.A C	3.547	THR73
				LEU 9 CB TYR 159.A CE1	3.967	TYR99
				SER 5 CA ARG 156.A HE	3.276	GLU152
				LEU 7 CD1 GLN 70.A CA	3.977	
LSPRWYFYY	−25.01	−24.40	−3.75	TYR 8 CE1 TYR 9.A OH	3.636	TYR9
				TRP 5 CB GLN 155.A C	3.962	ARG62
				TYR 9 C ARG 62.A HH11.B	3.082	ILE66
				PHE 7 CA ILE 66.A CD1	3.963	THR73
				LEU 1 CD1 GLN 155.A CG	3.965	TYR99
				PHE 7 O ILE 66.A CD1	3.568	GLU152
				TRP 5 CE3 GLN 155.A CB	3.857	

**FIGURE 2:**
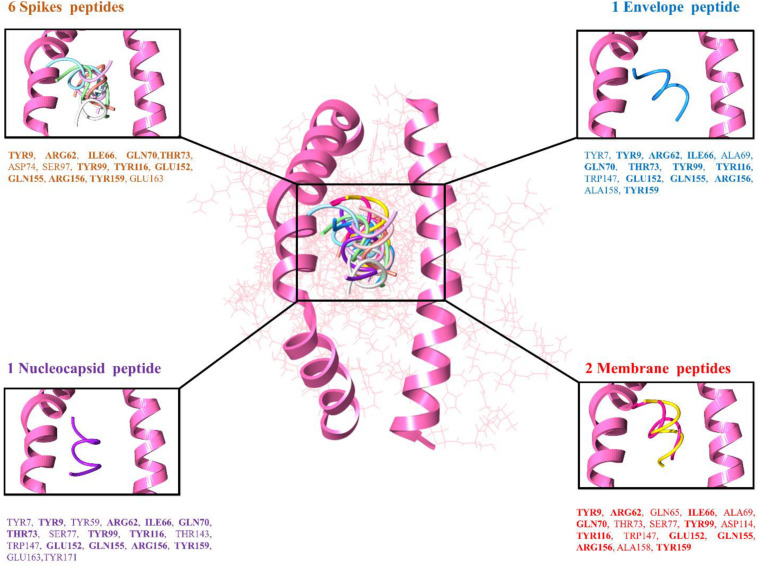
Peptide-MHC class I HLA-B (pink color helices denotes the conserved binding domain of HLA-B and the remaining protein structure is presented in the wire shape), binding interacting residues of the top-ranked 10 peptides represented in different colors, 6 spike peptides brown color residues, 2 membrane peptides red color residues, 1 nucleocapsid, and 1 envelope peptide with purple and blue color residues, respectively.

The top 10 docked complexes were visualized (Figure 2) and a similar binding pocket was observed in all the selected peptides. It was observed that Tyr9, Ile66, Gln70, Tyr99, Tyr116, and Arg156 residues were conserved in all the selected peptides (Table 3).

### Population Coverage Analyses

The population coverage analyses were performed with the selected MHC class I and MHC class II epitopes and also with the associated HLA alleles. It was observed that the selected MHC class I and MHC class II epitopes have the world’s population of 58.49 and 34.71%, respectively. MHC class I epitopes showed the highest coverage in the population of Italy (90.19%) and China (56.39%). The MHC class II epitopes also showed the highest coverage in the Philippines (71.92%) (Supplementary File 6).

### Multiple Sequence Alignment

Multiple sequence alignment was performed for three CoV genomes and conserved binding residues were observed. It was observed that all the selected strains of the CoV have conserved domains, which is reconciled with the latest outbreak strain SARS-CoV-2. Interestingly, it was observed that the reported binding domain of the previously reported strain has a similar region of binding with latest outbreak of CoV, 2019. The binding residues of SARS-CoV-2 showed similar binding domains with MERS and SARS (Supplementary File 7).

## Discussion

The need of dealing with CoVs has been increased since its recent breakout in China (Wuhan) affecting millions of humans. This SARS-CoV-2 viral attack has become a worldwide emergency in different regions of the World, especially in China (Mcclain, 1995). As an immediate response, numerous efforts from all over the world have been made to develop a peptide based vaccine against SARS-CoV-2, and the peptide inhibitors are of great interest to develop vaccines (Chew et al., 2017; Usman Mirza et al., 2017). The peptide targets are more preferable than traditional ligand-based drugs and vaccines due to different aspects including less toxic, fewer side-effects and their ultra-fast action. Immunoinformatics approaches help by reducing the work-load of laboratory trials, additionally these approaches are less time consuming and cost efficient than traditional approaches (Vanhee et al., 2011; Heurich et al., 2013; Xu et al., 2017). In the last 10 years, there has been much progress in *in silico* drug designing (Sehgal, 2017). Numerous biological problems are being solved by the implementation of different bioinformatics approaches (Sehgal et al., 2013; Sehgal, 2017; Tahir et al., 2018).

Researchers are striving mutually for a successful vaccine development and cure against COVID-19. Computational approaches were employed to analyze the synergistic effect by the combination of lopinavir, oseltamivir and ritonavir through molecular docking studies (Muralidharan et al., 2020).

Recently, molecular docking analyses along with virtual screening were performed against the drug candidates in clinical trials and approved drugs. Elbasvir, lopinavir, valrubicin, and carfilzomib were identified as potential compounds (Wang, 2020). Molecular docking analyses also revealed that luteolin and chloroquine also have the potential to inhibit the SARS-CoV-2 (Yu et al., 2020).

Recently, numerous research groups have struggled to design the subunit vaccines against SARS-CoV-2; though, the utilized workflow involved in the research either employ of a single protein to design the vaccine (Abdelmageed et al., 2020; Bhattacharya et al., 2020) or only CTL epitopes was used without considering the significance of HTL or B-cell epitopes (Seema, 2019). In current research work, all of these significant factors were considered to design the vaccine. Through extensive bioinformatics analyses, four proteins were utilized to design an epitope-based vaccine against SARS-CoV-2. The selected proteins for the analyses were membrane glycoprotein (M), nucleocapsid protein (N), envelop protein (E), and surface spike glycoprotein (S). The protein M helps in immunogenicity and assembly of the virus particles. The protein N has the ability to package the viral genome into a helical ribonucleocapsid and has a key role during viral self-assembly (Chang et al., 2013). The protein S has the ability to mediate the movement of the virus to human cells. The protein S is classified into two regions as S1 for the binding of the host receptor cell and S2 for the fusion of membrane. Due to the active involvement of protein S, it is considered as a key target for vaccine development, diagnostics and therapeutic antibodies for coronavirus (Du et al., 2009; Al-Amri et al., 2017; Prompetchara et al., 2020). By keeping the importance of protein S in mind, six different peptides were designed and analyzed.

The observed findings of antigenicity analysis range from 7.6 to 6.12% which is considered as an effective antigenic ability for a potent peptide, and similar ranges were observed in both studies of immunoinformatics analyses. Moreover, the binding domain of HLA-B was observed to be conserved in both studies and reconcile with the present research efforts (Usman Mirza et al., 2017; Tahir ul Qamar et al., 2020).

The potential CTL epitopes have been predicted for structural proteins of SARS-CoV-2. The molecular docking tools were used to analyze MHC-1 and peptide binding affinities for the selected peptides (Alam et al., 2016). Other evidences including C-terminal cleavage affinities also validated the binding affinity of the peptide-MHC-I complexes. In this study, ten peptides were reported as potential targets that showed effective interactions with the MHC-I protein (HLA-B), having maximum binding affinities and antigenicity. This increases the probability of the potential vaccine targets for the observed residues to be promising targets. The surface accessibility, surface flexibility as well as hydrophobicity and antigenicity for SARS-CoV-2 structural proteins were calculated and cross-verified by using the IEDB server (Sieker et al., 2009). An extensive literature review was performed and it was observed that the selected peptides were not reported against SARS-CoV-2. The predicted peptides were modeled by PEP-FOLD3 server and docked to MHC-1 using PatchDock and FireDock was used for further refinement. PyMOL and UCSF Chimera 1.11 were used to check the interactions of docked complexes.

The design and development of a potent vaccine needs an extensive investigation and analyses of immunological correlations with SARS-CoV-2. However, the experimental techniques would not be able to serve the urgency due to the severity and emergency of the COVID-19 outbreak. Therefore, *in silico* and computational predictions are helpful to guide the researchers to design a potential vaccine and help to control COVID-19. The vaccine development is an expensive and lengthy procedure with a high rate of failure, and several years are required to develop an effective commercial vaccine. Computational analyses suggest that the reported epitope-based vaccine peptides may have the ability to be protective against SARS-CoV-2 infection.

## Conclusion

The aim of this work was to identify the effective peptide based inhibitors against SARS-CoV-2 structural protein (Membrane, Envelope, Nucleocapsid, and Spikes). The predicted epitopes were designed leading to the molecular docking analyses against MHC-I and interactional analyses of the selected docked complexes were analyzed. In conclusion, 10 Epitopes (six from spikes protein “LTDEMIAQY, WTAGAAAYY, TSNQVAVLY, CVADYSVLY, KTSVDCTMY, and STECSNLLL,” two from membrane protein “SSDNIALLV and ATSRTLSYY,” one from nucleocapsid and one from envelope protein “LSPRWYFYY and LTALRLCAY,” respectively), were predicted which might be potential targets as peptide vaccine against deadly SARS -CoV-2.

## Data Availability Statement

The raw data supporting the conclusions of this article will be made available by the authors, without undue reservation, to any qualified researcher.

## Author Contributions

MW, AH, MS, SS, and SAS performed the analyses and drafted the manuscript. All authors contributed to the article and approved the submitted version.

## Conflict of Interest

The authors declare that the research was conducted in the absence of any commercial or financial relationships that could be construed as a potential conflict of interest.

## Footnotes

1 http://www.iedb.org/

2 http://tools.immuneepitope.org/toolsElliPro/

## Abbreviations

CTL: cytotoxic T-lymphocyte
HLA: human leukocyte antigen
IEDB: immune epitope database
MERS-CoV: middle east respiratory syndrome coronavirus
MHC: major histocompatibility complex
PI: isoelectric point
RBD: receptor binding domain
SARS-CoV: severe acute respiratory syndrome coronavirus.
