# Supplementary material for: Determine the Potential Epitope Based Peptide Vaccine Against Novel SARS-CoV-2 Targeting Structural Proteins Using Immunoinformatics Approaches
Source: Front Mol Biosci. 2020 Oct 15;7:227. doi: 10.3389/fmolb.2020.00227 (PMC7593713; doi:10.3389/fmolb.2020.00227)
Supplement: Supplementary file 5 [file Table_5.DOCX]

**Determine the potential Epitope based Peptide Vaccine against novel SARS-CoV-2 targeting structural proteins using immunoinformatics approach**

| Envelope | | | | | | |
| --- | --- | --- | --- | --- | --- | --- |
| **Residue Number** | **Peptide Sequence** | **Predicted MHC Binding Affinity** | **Rescale Binding Affinity** | **C-terminal Cleavage Affinity** | **TAP transport efficiency** | **Prediction score** |
| 34 | LTALRLCAY | 0.5594 | 2.3 | 0.672 | 2.933 | 2.6158 |
| 49 | VSLVKPSFY | 0.3533 | 1.499 | 0.3714 | 3.1 | 1.7149 |
| 54 | LVKPSFYVY | 0.1343 | 0.5702 | 0.9767 | 3.119 | 0.8726 |
| Membrane | | | | | | |
| **Residue Number** | **Peptide Sequence** | **Predicted MHC Binding Affinity** | **Rescale Binding Affinity** | **C-terminal Cleavage Affinity** | **TAP transport efficiency** | **Prediction score** |
| 213 | SSDNIALLV | 0.6531 | 2.7729 | 0.9682 | 0.286 | 2.9325 |
| 171 | ATSRTLSYY | 0.5463 | 2.3195 | 0.9375 | 3.09 | 2.6146 |
| 196 | YSRYRIGNY | 0.3214 | 1.3648 | 0.9345 | 3.148 | 1.6623 |
| 39 | YANRNRFLY | 0.3305 | 1.4031 | 0.4099 | 3.017 | 1.6155 |
| 170 | VATSRTLSY | 0.2752 | 1.1684 | 0.9679 | 3.013 | 1.4642 |
| 31 | WICLLQFAY | 0.2785 | 1.1825 | 0.5051 | 3.045 | 1.4105 |
| 87 | LVGLMWLSY | 0.2694 | 1.144 | 0.724 | 2.897 | 1.3974 |
| 188 | AGDSGFAAY | 0.1341 | 0.5695 | 0.9652 | 2.673 | 0.848 |
| 212 | SSSDNIALL | 0.1487 | 0.6313 | 0.9639 | 1.098 | 0.8308 |
| 16 | LLEQWNLVI | 0.1586 | 0.6734 | 0.5021 | 0.515 | 0.7745 |
| Nucleocapsid | | | | | | |
| **Residue Number** | **Peptide Sequence** | **Predicted MHC Binding Affinity** | **Rescale Binding Affinity** | **C-terminal Cleavage Affinity** | **TAP transport efficiency** | **Prediction score** |
| 104 | LSPRWYFYY | 0.4837 | 2.0538 | 0.9746 | 2.815 | 2.3408 |
| 164 | GTTLPKGFY | 0.3315 | 1.4074 | 0.9691 | 2.639 | 1.6848 |
| 103 | DLSPRWYFY | 0.2866 | 1.2167 | 0.976 | 2.725 | 1.4994 |
| 78 | SSPDDQIGY | 0.272 | 1.155 | 0.9463 | 3.144 | 1.4541 |
| 352 | LLNKHIDAY | 0.2587 | 1.0982 | 0.9685 | 2.863 | 1.3867 |
| 295 | GTDYKHWPQ | 0.2889 | 1.2267 | 0.0381 | -0.531 | 1.2059 |
| 79 | SPDDQIGYY | 0.204 | 0.8663 | 0.9769 | 2.551 | 1.1404 |
| 48 | NTASWFTAL | 0.1772 | 0.7523 | 0.9557 | 1.128 | 0.9521 |
| 101 | MKDLSPRWY | 0.1547 | 0.6569 | 0.7184 | 2.946 | 0.912 |
| Spikes | | | | | | |
| **Residue Number** | **Peptide Sequence** | **Predicted MHC Binding Affinity** | **Rescale Binding Affinity** | **C-terminal Cleavage Affinity** | **TAP transport efficiency** | **Prediction score** |
| 865 | LTDEMIAQY | 0.7953 | 3.3768 | 0.9723 | 2.779 | 3.6616 |
| 258 | WTAGAAAYY | 0.6735 | 2.8596 | 0.7339 | 2.863 | 3.1128 |
| 604 | TSNQVAVLY | 0.6559 | 2.7847 | 0.944 | 2.991 | 3.0758 |
| 361 | CVADYSVLY | 0.5348 | 2.2705 | 0.9764 | 3.18 | 2.5759 |
| 733 | KTSVDCTMY | 0.4908 | 2.084 | 0.9649 | 3.016 | 2.3795 |
| 746 | STECSNLLL | 0.5136 | 2.1808 | 0.8879 | 0.703 | 2.3492 |
| 652 | GAEHVNNSY | 0.4042 | 1.7163 | 0.9769 | 2.663 | 1.996 |
| 196 | NIDGYFKIY | 0.3921 | 1.6649 | 0.9664 | 3.015 | 1.9606 |
| 160 | YSSANNCTF | 0.3975 | 1.6878 | 0.9032 | 2.598 | 1.9531 |
| 152 | WMESEFRVY | 0.3902 | 1.6569 | 0.7993 | 2.929 | 1.9232 |
| 162 | SANNCTFEY | 0.3737 | 1.5865 | 0.9196 | 2.99 | 1.8739 |
| 687 | VASQSIIAY | 0.3529 | 1.4986 | 0.9656 | 3.089 | 1.7978 |
| 30 | NSFTRGVYY | 0.3389 | 1.4389 | 0.6421 | 3.124 | 1.6915 |
| 136 | CNDPFLGVY | 0.2613 | 1.1095 | 0.69 | 2.45 | 1.3355 |
| 392 | FTNVYADSF | 0.2704 | 1.148 | 0.38 | 2.317 | 1.3208 |
| 261 | GAAAYYVGY | 0.2253 | 0.9568 | 0.7608 | 2.969 | 1.2194 |
| 357 | RISNCVADY | 0.2106 | 0.8941 | 0.9292 | 3.394 | 1.2032 |
| 465 | ERDISTEIY | 0.2097 | 0.8903 | 0.9744 | 2.646 | 1.1687 |
| 285 | ITDAVDCAL | 0.235 | 0.9979 | 0.8708 | 0.79 | 1.168 |
| 1039 | RVDFCGKGY | 0.2036 | 0.8644 | 0.7618 | 3.232 | 1.1403 |
| 343 | NATRFASVY | 0.1955 | 0.83 | 0.9342 | 2.873 | 1.1138 |
| 1237 | MTSCCSCLK | 0.226 | 0.9595 | 0.7525 | 0.479 | 1.0963 |
| 50 | STQDLFLPF | 0.1974 | 0.8383 | 0.553 | 2.511 | 1.0468 |
| 1096 | VSNGTHWFV | 0.2012 | 0.8544 | 0.6143 | 0.218 | 0.9574 |
| 880 | GTITSGWTF | 0.1656 | 0.7031 | 0.7489 | 2.557 | 0.9433 |
| 815 | RSFIEDLLF | 0.1421 | 0.6035 | 0.5938 | 3.032 | 0.8441 |
| 1264 | VLKGVKLHY | 0.1262 | 0.5356 | 0.9783 | 2.859 | 0.8253 |
| 748 | ECSNLLLQY | 0.1413 | 0.6 | 0.5316 | 2.747 | 0.8171 |
| 370 | NSASFSTFK | 0.1671 | 0.7093 | 0.5456 | 0.507 | 0.8165 |
| 372 | ASFSTFKCY | 0.118 | 0.501 | 0.9587 | 3.275 | 0.8085 |
| 628 | QLTPTWRVY | 0.1189 | 0.5047 | 0.9661 | 2.782 | 0.7887 |
| 296 | LSETKCTLK | 0.1515 | 0.6432 | 0.8919 | 0.22 | 0.7879 |
| 192 | FVFKNIDGY | 0.1358 | 0.5767 | 0.4093 | 2.913 | 0.7837 |
| 445 | VGGNYNYLY | 0.1164 | 0.4941 | 0.9518 | 2.658 | 0.7698 |
| 83 | VLPFNDGVY | 0.113 | 0.4797 | 0.9703 | 2.846 | 0.7675 |
| 1095 | FVSNGTHWF | 0.1232 | 0.5231 | 0.7203 | 2.621 | 0.7622 |
| 612 | YQDVNCTEV | 0.1531 | 0.6501 | 0.587 | 0.242 | 0.7502 |
