# Supplementary material for: Determine the Potential Epitope Based Peptide Vaccine Against Novel SARS-CoV-2 Targeting Structural Proteins Using Immunoinformatics Approaches
Source: Front Mol Biosci. 2020 Oct 15;7:227. doi: 10.3389/fmolb.2020.00227 (PMC7593713; doi:10.3389/fmolb.2020.00227)
Supplement: Supplementary file 6 [file Table_6.DOCX]

**Determine the potential Epitope based Peptide Vaccine against novel SARS-CoV-2 targeting structural proteins using immunoinformatics approach**

**Table4:** Populations coverage analysis country-wise coverage prediction for MHC-Class I and MHC-Class II.

| **Population/area** | **Class I** | | | **Class II** | | |
| --- | --- | --- | --- | --- | --- | --- |
|  | **Coverage a** | **average_hitb** | **pc90c** | **coveragea** | **average_hitb** | **pc90c** |
| [**Belgium**](http://tools.iedb.org/population/result/#Belgium) | 57.86 | 2.13 | 0.24 | 47.15 | 1.57 | 0.38 |
|  |  |  |  |  |  |  |
| [**Brazil**](http://tools.iedb.org/population/result/#Brazil) | 71.88 | 5.32 | 0.36 | 24.62 | 0.82 | 0.27 |
|  |  |  |  |  |  |  |
| [**China**](http://tools.iedb.org/population/result/#China) | 56.39 | 4.02 | 0.23 | 24.62 | 0.91 | 0.27 |
|  |  |  |  |  |  |  |
| [**Finland**](http://tools.iedb.org/population/result/#Finland) | 89.59 | 7.57 | 0.96 | 20.08 | 0.47 | 0.25 |
|  |  |  |  |  |  |  |
| [**France**](http://tools.iedb.org/population/result/#France) | 86.01 | 7.31 | 0.71 | 5.08 | 1.65 | 0.41 |
|  |  |  |  |  |  |  |
| [**Germany**](http://tools.iedb.org/population/result/#Germany) | 86.19 | 7.06 | 0.72 | 50.44 | 1.69 | 0.4 |
|  |  |  |  |  |  |  |
| [**Hong Kong**](http://tools.iedb.org/population/result/#Hong%20Kong) | 42.46 | 3.44 | 0.35 | 0 | 0 | 0 |
|  |  |  |  |  |  |  |
| [**India**](http://tools.iedb.org/population/result/#India) | 67.07 | 5.14 | 0.3 | 53.05 | 2.07 | 0.43 |
|  |  |  |  |  |  |  |
| [**Indonesia**](http://tools.iedb.org/population/result/#Indonesia) | 4.65 | 3.33 | 0.37 | 59.56 | 2.44 | 0.74 |
|  |  |  |  |  |  |  |
| [**Iran**](http://tools.iedb.org/population/result/#Iran) | 78.28 | 6.86 | 0.46 | 4.51 | 1.75 | 0.36 |
|  |  |  |  |  |  |  |
| [**Italy**](http://tools.iedb.org/population/result/#Italy) | 90.19 | 8.43 | 1.37 | 20.84 | 0.63 | 0.25 |
|  |  |  |  |  |  |  |
| [**Japan**](http://tools.iedb.org/population/result/#Japan) | 77.57 | 7.68 | 0.89 | 24.89 | 0.95 | 0.27 |
|  |  |  |  |  |  |  |
| [**Korea; South**](http://tools.iedb.org/population/result/#Korea;%20South) | 76.35 | 7.05 | 0.85 | 27.01 | 0.98 | 0.27 |
|  |  |  |  |  |  |  |
| [**Malaysia**](http://tools.iedb.org/population/result/#Malaysia) | 4.68 | 2.91 | 0.38 | 38.85 | 1.56 | 0.49 |
|  |  |  |  |  |  |  |
| [**Netherlands**](http://tools.iedb.org/population/result/#Netherlands) | 0 | 0 | 0 | 43.63 | 1.49 | 0.35 |
|  |  |  |  |  |  |  |
| [**New Zealand**](http://tools.iedb.org/population/result/#New%20Zealand) | 0 | 0 | 0 | 43.89 | 1.61 | 0.36 |
|  |  |  |  |  |  |  |
| [**Pakistan**](http://tools.iedb.org/population/result/#Pakistan) | 52.83 | 3.06 | 0.21 | 0 | 0 | 0 |
|  |  |  |  |  |  |  |
| [**Philippines**](http://tools.iedb.org/population/result/#Philippines) | 60.01 | 4.57 | 0.5 | 71.92 | 3.05 | 1.07 |
|  |  |  |  |  |  |  |
| [**Russia**](http://tools.iedb.org/population/result/#Russia) | 77.25 | 6.73 | 0.44 | 39.31 | 1.23 | 0.33 |
|  |  |  |  |  |  |  |
| [**Singapore**](http://tools.iedb.org/population/result/#Singapore) | 56.52 | 4.41 | 0.46 | 4.25 | 1.67 | 0.47 |
|  |  |  |  |  |  |  |
| [**Spain**](http://tools.iedb.org/population/result/#Spain) | 68.78 | 5.16 | 0.32 | 52.23 | 1.82 | 0.42 |
|  |  |  |  |  |  |  |
| [**Sudan**](http://tools.iedb.org/population/result/#Sudan) | 84.61 | 6.6 | 0.65 | 45.39 | 1.65 | 0.37 |
|  |  |  |  |  |  |  |
| [**Sweden**](http://tools.iedb.org/population/result/#Sweden) | 83.44 | 5.47 | 1.21 | 35.38 | 1.14 | 0.31 |
|  |  |  |  |  |  |  |
| [**Taiwan**](http://tools.iedb.org/population/result/#Taiwan) | 60.41 | 5.09 | 0.25 | 25.23 | 1.01 | 0.27 |
|  |  |  |  |  |  |  |
| [**Thailand**](http://tools.iedb.org/population/result/#Thailand) | 59.51 | 3.87 | 0.25 | 44.36 | 1.77 | 0.36 |
|  |  |  |  |  |  |  |
| [**Turkey**](http://tools.iedb.org/population/result/#Turkey) | 23.09 | 1.15 | 0.65 | 48.45 | 1.84 | 0.39 |
|  |  |  |  |  |  |  |
| [**United Arab Emirates**](http://tools.iedb.org/population/result/#United%20Arab%20Emirates) | 2.19 | 0.04 | 0.2 | 0 | 0 | 0 |
|  |  |  |  |  |  |  |
| [**United Kingdom**](http://tools.iedb.org/population/result/#United%20Kingdom) | 44.55 | 2.13 | 0.47 | 0 | 0 | 0 |
|  |  |  |  |  |  |  |
| [**Vietnam**](http://tools.iedb.org/population/result/#Vietnam) | 49.77 | 3.68 | 0.4 | 27.17 | 1.04 | 0.27 |
|  |  |  |  |  |  |  |
| **Average** | 58.49 | 4.49 | 0.49 | 34.71 | 1.27 | 0.34 |
| **Standard deviation** | 25.4 | 2.38 | 0.32 | 18.39 | 0.73 | 0.21 |
