# Supplementary material for: Determine the Potential Epitope Based Peptide Vaccine Against Novel SARS-CoV-2 Targeting Structural Proteins Using Immunoinformatics Approaches
Source: Front Mol Biosci. 2020 Oct 15;7:227. doi: 10.3389/fmolb.2020.00227 (PMC7593713; doi:10.3389/fmolb.2020.00227)

**Determine the potential Epitope based Peptide Vaccine against novel SARS-CoV-2 targeting structural proteins using immunoinformatics approach**

**Multiple Sequence Alignment**


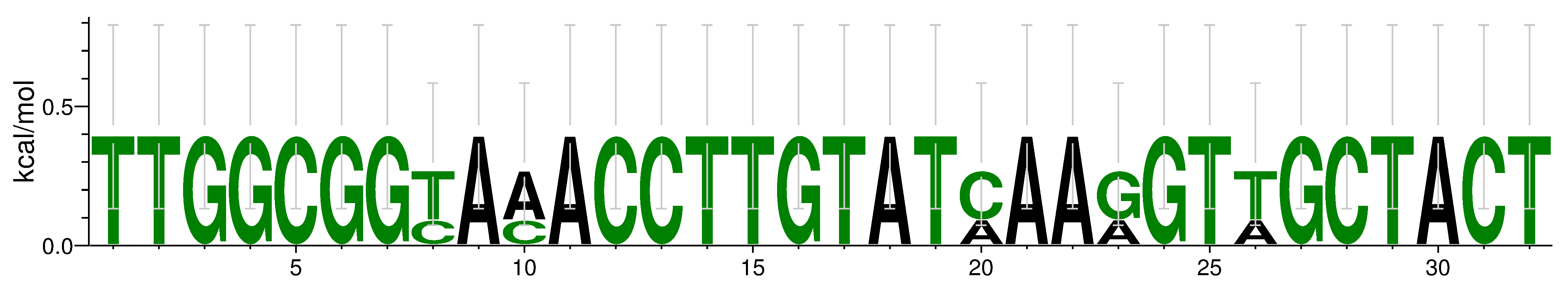
**Figure 1:** Highly conserved sequence among all three genomes.


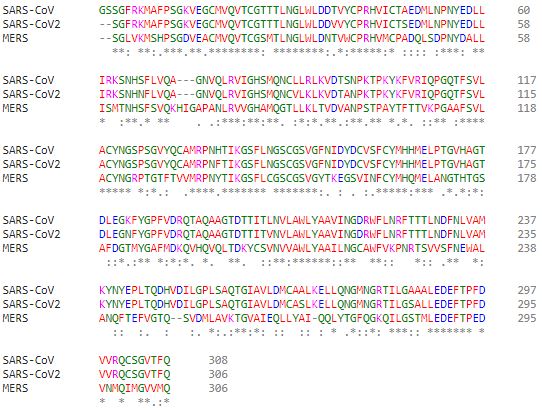


**Figure 2:** MSA of all three nonstructural proteins (6lu7 from SARS-CoV2, 4rsp from MERS and 3m3v from SARS-CoV) using Clustal Omega to check conserved domain sequence.

**Classification:**


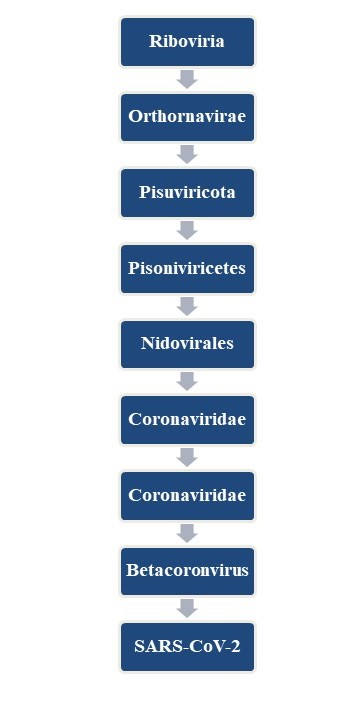

Supplement: Supplementary file 7 [file Table_7.DOCX]
