# Supplementary material for: Determine the Potential Epitope Based Peptide Vaccine Against Novel SARS-CoV-2 Targeting Structural Proteins Using Immunoinformatics Approaches
Source: Front Mol Biosci. 2020 Oct 15;7:227. doi: 10.3389/fmolb.2020.00227 (PMC7593713; doi:10.3389/fmolb.2020.00227)
Supplement: Supplementary file 8 [file Table_8.DOCX]

**Determine the potential Epitope based Peptide Vaccine against novel SARS-CoV-2 targeting structural proteins using immunoinformatics approach**

Table 16: Physiochemical properties of structural proteins using VOLPES <http://volpes.univie.ac.at/>.

| **Protein** | **Length** | **Molecular Weight**  **(g/mol)** | **Hydropathicity** | **Theoretical PI** | **Max. surface accessibility**  **(%)** | **Min. surface accessibility**  **(%)** | **Max. Flexibility**  **(%)** | **Min. Flexibility**  **(%)** | **Max. Antigenicity**  **(%)** | **Min. Antigenicity**  **(%)** |
| --- | --- | --- | --- | --- | --- | --- | --- | --- | --- | --- |
| Membrane | 222 | 25146.6 | 0.456 | 9.51 | 5.109 | 1.078 | 8.725 | 0.061 | 7.116 | 0.01 |
| Envelope | 75 | 83650.4 | 1.12 | 8.57 | 3.136 | 0.078 | 6.513 | 0.015 | 8.5 | 0.002 |
| Nucleocapsid | 419 | 45625.7 | -0.871 | 10.07 | 6.956 | 0.05 | 7.8 | 0.052 | 6.95 | 0.05 |
| Spikes | 1273 | 141178 | -0.069 | 6.24 | 6.05 | 0.17 | 5.62 | 1.3 | 7.121 | 0.0751 |
