# Supplementary material for: Determine the Potential Epitope Based Peptide Vaccine Against Novel SARS-CoV-2 Targeting Structural Proteins Using Immunoinformatics Approaches
Source: Front Mol Biosci. 2020 Oct 15;7:227. doi: 10.3389/fmolb.2020.00227 (PMC7593713; doi:10.3389/fmolb.2020.00227)
Supplement: Supplementary file 9 [file Table_9.docx]

**Determine the potential Epitope based Peptide Vaccine against novel SARS-CoV-2 targeting structural proteins using immunoinformatics approach**

**BLASTp**

Database: non-redundant protein sequences

Expect threshold: 10

Matrix: BLOSUM62

Gap costs: Existence, 11 extension, 1

Compositional adjustments: Conditional compositional score matrix adjustment

**For BLASTn (for whole genomes)**

Database: non-redundant nucleotide collection

Expect threshold: 10

Match-mismatch: 1, -2

Gap costs: linear

**Clustal omega (MSA)**

Max guide tree iterations: 2

Max HMM iterations: 3

Number of Combined iterations: 5

**Weblogo3: default**

**ABCpred (B cell epitopes)**

Threshold: 0.51

Window size: 16

Overlapping: on

**NetCTL1.2 server**

Supertype: A1 supertype

Weight on C terminal cleavage: 0.15

Weight on TAP transport efficiency: 0.05

Threshold for epitope identification: 0.75

**ElliPro (Antibody Epitope Prediction)**

Minimum score: 0.5

Maximum distance: 6 angstrom

**Population coverage analysis**

This allele set is used: HLA-DRB1*03:01

HLA-DRB1*07:01

HLA-DRB1*15:01

HLA-DRB3*01:01

HLA-DRB3*02:02

HLA-DRB4*01:01

HLA-DRB5*01:01

HLA-DRB1*01:01

HLA-DRB1*03:01

HLA-DRB1*04:01

HLA-DRB1*04:05

HLA-DRB1*07:01

HLA-DRB1*08:02

HLA-DRB1*09:01

HLA-DPA1*02:01/DPB1*14:01

- All properties such as Bepipred Linear Epitope Prediction, Chou & Fasman Beta-Turn Prediction, Emini Surface Accessibility Prediction, Karplus & Schulz Flexibility Prediction, Kolaskar & Tongaonkar Antigenicity, Parker Hydrophilicity Prediction all were predicted at default parameters.

**Toxinpred (toxicity)**

SVM method applied

E-value: 10

Threshold: 0.5

**Vexijen (antigenicity)**

Threshold: 0.5

**PEP-FOLD3**

Simulations: 200

Energy Function: sOPEP

**PATCHDOCK**

RMSD: 4.0

Distance constraints: default

**FIREDOCK**

Refinement level: restricted

Number of RBO Cycles: 50

Atomic radius scale: 0.8

Final selection of top ranked 10 epitopes for peptide designing and molecular docking analyses were based upon manual identification. All results including surface properties, Antigenicity, Toxicity, and Immunogenicity were manually analyzed to select most efficient epitopes.
